# The Effect of Cooled Perches on Immunological Parameters of Caged White Leghorn Hens during the Hot Summer Months

**DOI:** 10.1371/journal.pone.0141215

**Published:** 2015-10-23

**Authors:** Rebecca A. Strong, Patricia Y. Hester, Susan D. Eicher, Jiaying Hu, Heng-Wei Cheng

**Affiliations:** 1 Department of Animal Sciences, Purdue University, West Lafayette, Indiana, United States of America; 2 Untied States Department of Agriculture, Livestock Behavior Research Unit, West Lafayette, Indiana, United States of America; King Abdullah International Medical Research Center, SAUDI ARABIA

## Abstract

The objective of this study was to determine if thermally cooled perches improve hen immunity during hot summer. White Leghorn pullets at 16 week of age were randomly assigned to 18 cages of 3 banks at 9 hens per cage. Each bank was assigned to 1 of the 3 treatments up to 32 week of age: 1) thermally cooled perches, 2) perches with ambient air, and 3) cages without perches. Hens were exposed to natural ambient temperatures from June through September 2013 in Indiana with a 4 h acute heat episode at 27.6 week of age. The packed cell volume, heterophil to lymphocyte (H/L) ratio, plasma concentrations of total IgG, and cytokines of interleukin-1β and interleukin-6, plus lipopolysaccharide-induced tumor necrosis factor-α factor were measured at both 27.6 and 32 week of age. The mRNA expressions of these cytokines, toll-like receptor-4, and inducible nitric oxide synthase were also examined in the spleen of 32 week-old hens. Except for H/L ratio, thermally cooled perches did not significantly improve currently measured immunological indicators. These results indicated that the ambient temperature of 2013 summer in Indiana (24°C, 17.1 to 33.1°C) was not high enough and the 4 h heat episode at 33.3°C (32 to 34.6°C) was insufficient in length to evoke severe heat stress in hens. However, cooled perch hens had a lower H/L ratio than both air perch hens and control hens at 27.6 week of age and it was still lower compared to control hens (*P* < 0.05, respectively) at 32 week of age. The lowered H/L ratio of cooled perch hens may suggest that they were able to cope with acute heat stress more effectively than control hens. Further studies are needed to evaluate the effectiveness of thermally cooled perches on hen health under higher ambient temperatures.

## Introduction

During summer, high ambient temperature is one of the most important environmental stressors adversely affecting poultry health [1–2]. Hens to loss heat are limited due to feathering and the absence of sweat glands [1]. A hen can tolerate and adapt to ambient temperatures up to 25°C (77°F); temperatures above this level can lead to heat stress (**HS**) as a combination of hen’s physical heat production plus the environmental heat load is greater than its ability to lose heat, leading to an increase in body core temperature, and possibly death [3–4]. Especially during heat waves without acclimation [5–6], a chicken can die suddenly of heat stroke. In the Midwest, for example, the exceptionally hot summers of 1995, 2011, and 2012 were devastating to the egg industry as hen mortalities climbed to 10% due to heat [4]. For those hens which survive high temperatures, HS reduces their body weight gain [7–8], feed intake [9–10], egg production [11–12], and egg quality [13–15]. Currently, with climate change and global warming, chicken growers are struggling to fight summer heat.

It is well established that high environmental temperatures also affect immune function in hens, causing immunosuppression [16–19]. The degree of heat-induced immunosuppression depends on multiple factors including the length and intensity of the heat exposure [10, 20–21]. In hens, HS limits immunocompetence by suppressing antibody production [8] and alters the populations of immune cells, leading to an increase in the heterophil to lymphocyte (**H/L**) ratio which has been used as an indicator of stress [22–25]. With hens having only rudimentary lymph nodes [26–27], they rely on the spleen as a major immune organ as it is an important site for immune responses to antigens [28–29]. Hens with greater spleen weights have higher immunocompetence [30]. In addition, spleen weight decreased in laying hens [10, 31] and broilers [19, 32] after exposure to high temperatures. An additional indicator of stress besides lymphoid organ regression is the production of cytokines [33]. As molecular messengers, cytokines are involved in cell signaling and are synthesized by a variety of cells including monocytes, and B- and T-lymphocytes [34–37]. Cytokines play an important role in immunity by coordinating the humoral (B-cell) and cell mediated immune (T-cell) responses [38–42]. Examples of cytokines include the interleukin (**IL**) family and lipopolysaccharide-induced tumor necrosis factor-α factor (**LITAF**, similar to TNF-α in mammals) [43] produced by several immune cells including the CD4 T-helper cells [44]. The IL-1 has a key role in the inflammatory response and stimulates B- and T-cell development and differentiation [45–49]. The TNF-α is synthesized by monocytes and acts as a cytotoxin promoting apoptosis leading to tumor regression [50]. In addition to cytokines, toll-like receptor (**TLR**)-4 is a protein receptor found on the membranes of macrophages and dendritic cells [51]. These receptors recognize conserved sections of invading antigens to trigger the activation of immune cells [52]. In addition, a non-specific immune defense mechanism that hens may use during stressful conditions is nitric oxide. Nitric oxide is generated by phagocytes as part of the immune response [53–54]. Because of an unpaired electron, the nitric oxide produced by the inducible isoform of nitric oxide synthase (**iNOS**) acts as a free radical, attacking and destroying antigens such as viruses, bacteria, tumors, and parasites [55]. Nitric oxide also influences inflammatory responses [56]. There are many cell types that synthesize iNOS in response to cytokines [57–58]. Packed cell volume (**PCV**), the volume percentage of blood cells in blood, is another health indicator of humans and animals [59–61] including chickens exposed to HS [24, 62]. Attia et al. [62] reported that PCV was reduced in broiler chickens followed chronic HS, which was correlated with increased rectal temperature and respiration rate.

The majority of hens are currently housed in conventional cages in the United States; however, egg producers are updating their facilities with cages that can eventually be enriched with a nest, perches, scratch pad, and nail trim area [63–65]. Introducing perches stimulates a variety of motor patterns [66] with perching causing an increase in bone strength in laying hens [67–69]. Allowing access to cooled perches during the summer hot months may help alleviate HS for laying hens, because hens have a natural tendency to perch for resting and protection [70] and more than 25% of the heat produced by hens can be lost through their feet by modulating blood flow in hot environments [71]. Increased conductive heat transfer from the feet to a thermally controlled perch helps chickens to relieve HS as that broiler breeder hens [72] and broiler chickens [73] improved growth performance with access to cooled perches during high temperatures [73–74]. Broiler chickens subjected to HS with access to cooled perches exhibited less panting and had reduced core body temperatures compared to controls [75]. Cooled perch availability increased body weight gain and feed efficiency of broiler chickens in high ambient temperatures regardless of stocking density [76].

Currently, there are no studies on cooled perches that evaluate the immune responses of laying hens when exposed to high ambient temperatures. Therefore, the aim of the present study was to determine if cooled perches inhibit heat stress-induced immunological suppression in caged laying hens. Our hypothesis was that hens with cooled perches during hot weather will exhibit improved well-being as decreased H/L ratios, adrenal weights, and cytokines; down-regulation of splenic cytokines, TLR-4, and iNOS; and greater body weight, spleen weights, and plasma IgG concentrations as compared to hens without access to cooled perches.

## Materials and Methods

### Birds and Management

One-hundred and sixty-two Hy-Line W36 White Leghorn pullets, 16 week of age, were transported to the Layer Research Unit at Purdue University’s Poultry Research Farm located in West Lafayette of Indiana. The pullets were individually weighed and 162 chickens with similar body weight were leg tagged and randomly assigned to 3 banks with 18 laying cages at 9 hens per cage for 16 weeks. Within a bank, there were 3 tiers with 2 cages per tier. Feeder and floor space were 8.4 cm and 439 cm^2^ per hen, respectively. Two nipple drinkers were assigned to each laying cage. Dropping boards were located between tiers of cages.

A pre-lay diet with calculated values of 3,009 kcal ME/ kg, 20.0% CP, 1.0% Ca, and 0.45% non-phytate P was fed from 16 to 17 week of age. At 17 week of age, chickens consumed a layer diet with 2,890 kcal ME/kg, 18.3% CP, 4.2% Ca, and 0.30% non-phytate P until the end of the study at 32 week of age. Throughout the study, hens had free access to feed and water.

At 16 week of age, light hours were gradually stepped up from 12 h achieving a photoperiod of 16L:8D by 30 week of age, where it remained until termination of the study. Light intensity was set at 10.7 lux.

The protocol was approved by the Purdue University Animal Care and Use Committee.

### Treatments

**Fig 1:**
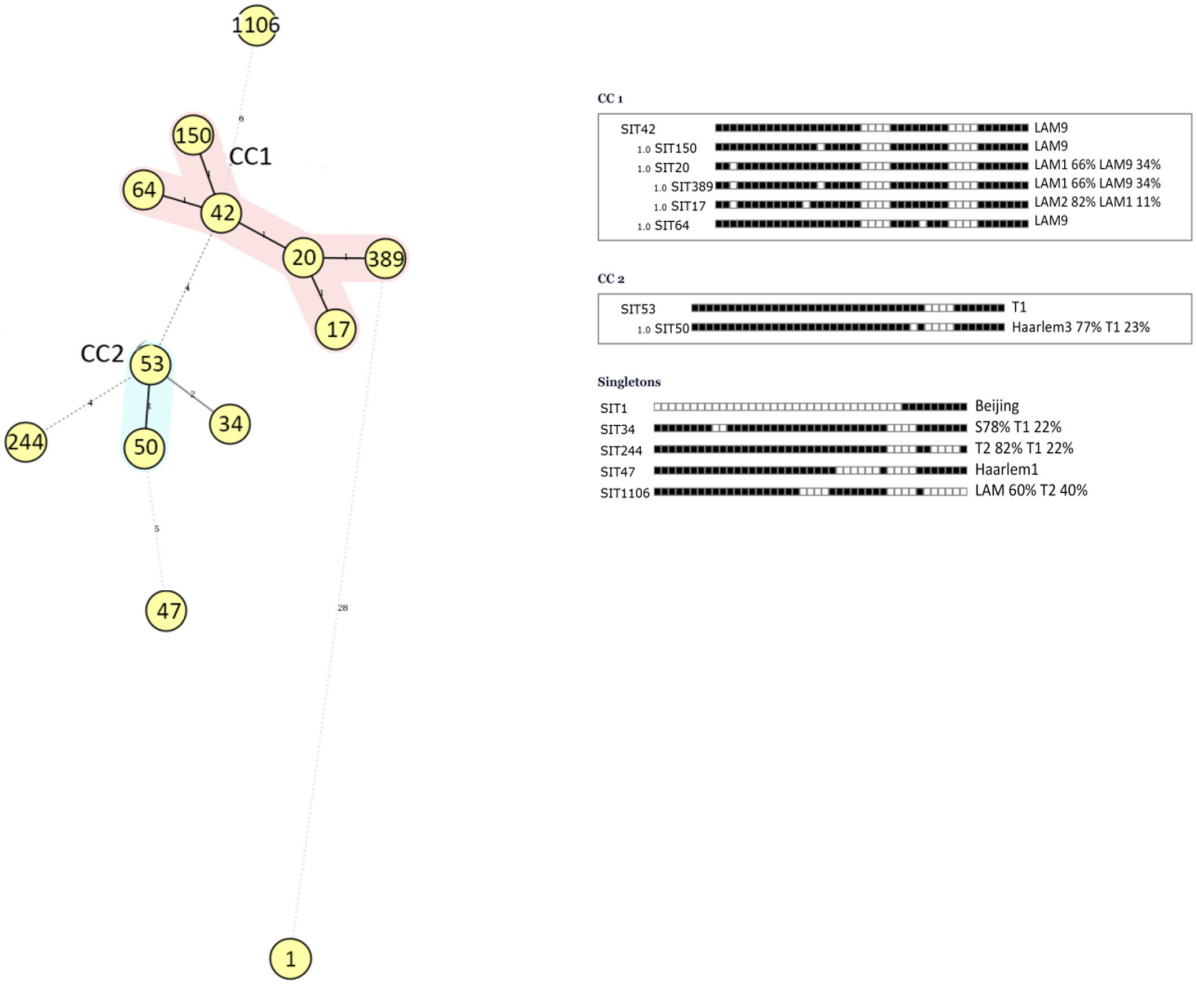
The bank of cages assignment for the thermally cooled perch treatment. Each tier had its own pump to distribute chilled deionized water (10°C) through its perch loop that ran parallel to the feeder. The front perch closest to the feeder received chilled water pumped directly from a common vertical manifold. The back perch was the return loop that sent the water back to the common manifold to be re-chilled. A chiller was used to cool the water in the manifold; it had its own independent thermostat which kept the water at 10°C. A separate 4th pump continuously circulated the deionized water between the water chiller and the manifold. A sensor for monitoring air temperature was installed to the controller of each tier to activate or stop the circulation of chilled water through the perch loop when ambient temperature reached or fell below 25°C, respectively [75].

Each bank was assigned randomly to 1 of the following treatments: (1) thermally cooled perches, (2) perches with ambient air, and (3) conventional cages without perches (control). For the bank of cages assigned the cooled perch treatment, each tier had its own pump to distribute chilled deionized water (10°C) through its perch loop, round galvanized steel pipes (3.38 cm outside diameter), that ran parallel to the feeder (Fig 1). This looped arrangement provided 2 perches per cage giving each hen 16.9 cm of perch space. The cage dimensions and perch placement within the laying cages were reported previously [77]. The front perch closest to the feeder received chilled water pumped directly from a common vertical manifold. The back perch was the return loop that sent the water back to the common manifold to be re-chilled. A chiller was used to cool the water in the manifold; it had its own independent thermostat which kept the water at 10°C. A separate 4th pump continuously circulated the deionized water between the water chiller and the manifold. A sensor for monitoring air temperature was installed to the controller of each tier to activate or stop the circulation of chilled water through the perch loop when ambient temperature reached or fell below 25°C, respectively. The air perches were also arranged in a loop system as described for the cooled perches, but no water was pumped through these perches. Air temperature and relative humidity within the room, the cage, the water temperature of supply and return lines of the cooled perches, and the air temperature of the air perches were recorded using a wireless monitoring system [78].

To imitate poultry production condition and to identify the effects of thermally cooled perch system in prevent HS, evaporative cooling pads were not used at any time during the study to allow exposure of hens to naturally hot summer days. The study was conducted from June through September 2013 in Indiana of the United States. Environmental condition inside the house were not controlled throughout the study with the exception of an acute heating episode where the ambient temperature was elevated to a mean of 33.3°C for 4 h at 27.6 week of age by providing auxiliary heat. The heating episode was initiated 9 h following initiation of photo-stimulation after most hens had laid their eggs for that day.

### Sampling

Two hens without an egg in their uterus as determined through palpation were randomly taken from each cage for sampling at 27.6 week of age (n = 12 per treatment). To minimize effects of circadian variation on the measured parameters, blood samples were collected from a hen per cage of each treatment by repeating the cycle of cooled perch, air perch and no perch until the end. The sample collection began at 2 h post initiation of the heating episode to ensure all samples were collected within the 4 h acute heat stress period. A 4-mL blood sample was collected from each bird via the brachial vein within 2 min of being handled. The blood was collected into EDTA-coated test tubes. A leg ring was placed on the right shank before returning the hen to its cage.

At 32 week, 2 hens per cage (n = 12 per treatment) without a leg ring were randomly removed from their cage and sampled using the order of treatments as described for the collection of blood at 27.6 week of age. Hens were sedated by injecting 30 mg of sodium pentobarbital/kg of body weight into the brachial vein. A 5-mL blood sample was collected from each hen by cardiac puncture, and the blood placed into EDTA-coated test tubes. The hen was killed by cervical dislocation immediately followed blood sampling; and its body weight was recorded. The spleen, liver, and the right adrenal gland of each sampled hen were collected and weighed. The spleen was stored at -80°C until further analysis.

All blood samples were stored on ice and transported to the laboratory to be centrifuged at 700 x g for 20 min at 4°C. The supernatant plasma was collected and stored at -80°C until analysis.

### Quantitative Analysis of Blood Parameters

#### PCV

Heparinized glass capillary tubes were used to collect blood from a venipuncture of the brachial vein of the 2 hens that were used in blood collection at 27.6 and 32 week age. The duplicate hematocrits were spun for 15 min and the proportion of the total volume making up the cells was determined.

#### H/L Ratio

Immediately following the collection of hematocrits, duplicate blood smears per hen were made by generating a thin layer of cells along the slide at 27.6 and 32 week of age, respectively. After drying, the slides were stained with Wright’s staining. Through light microscopy, heterophils and lymphocytes were differentiated based on a previous counting method on the single layer cell area within the meddle 3/5 of each slide [79]. A total of 100 heterophil and lymphocyte per slide or a total 200 cells per hen per time point were counted, and then the H/L ratio was calculated [80].

### ELISA

Commercially available chicken specific ELISA kits were used to measure chicken plasma IgG (Catalog No. E33-104; Bethyl Laboratories, Inc., Montgomery, TX) and cytokines of IL-1β (product code: CSB-E11230Ch. American Research Products, Inc., Waltham, MA), IL-6 (product code: CSB-E08549Ch), and LITAF (product code: CSB-E11231Ch) using the instructions from the respective companies. Briefly, plasma samples were diluted to 1:1000 with supplied sample buffer for IgG (1X Dilution Buffer B), the rest of the parameters were tested in original concentration, all samples were analyzed in duplicate with an absorbance reading of 450 nm by following the manufacture’s protocols and methods reported previously [81].

### Gene Expression

Spleen mRNA expression of IL-1β, IL-6, LITAF, iNOS, and TLR-4 (Applied Biosystems, Life Technologies, Grand Island, NY) were detected by real-time PCR using the primers and probes (Table 1) developed elsewhere [10]. Results were quantitated by standard curve method, and data were log transferred and expressed as relative abundance of the interested genes to the reference gene glyceraldehyde 3-phosphate dehydrogenase (GAPDH) following the method described previously [10]. Briefly, after RNA extraction, reverse transcription was conducted using 61.5 μL of master mix, made of 2.5μL of Multi-Scribe reverse transcriptase, 22 μL of 25 mM MgCl, 5 μl random hexamers, 2μL RNase inhibitor, 20 μl dNTPs, and 10 μL of TaqMan reverse transcription buffer provided in the TaqMan Reverse Transcription Reagent Pack (Applied Biosystems, Foster City, CA). The 61.5 μL of master mix was then added to the quantified RNA sample and RNase-free water (Ambion Inc.) for a total of 100 μL. Reverse transcription and amplification was done using the Hybaid PCR Express thermo cycler (Midwest Scientific, St. Louis, MO). Stock primers and probes were diluted to 10 μM solutions. The conditions for PCR were a ratio of 1.625 μL of TaqMan probe, 2.25 μL of gene- specific TaqMan forward and reverse primers each, 12.5 μL of PCR Mastermix (Applied Biosystems), 3.875 μL RNase-free water (Ambion Inc.), and 2.5 μL of sample cDNA. Standards were measured in triplicates and samples in duplicates with a standard deviation of less than 2.0 and a coefficient of variation less than 2.0% for all spleen mRNA expression.

**Table 1:** Taqman primers and probes used.

Gene	Primers and Probe (5'-3')	Application Efficiencies (%)	Product Length (bp)	Reference/ Accession no.
IL-1β	(f) TGCTGGTTTCCATCTCGTATGTAC (r) CCCAGAGCGGCTATTCCA (p) AGTACAACCCCTGCTGCCCCGC (VIC/MGB)	95	80	NC_006096.3
IL-6	(f) CCCGCTTCTGACTGTGTTT (r) GCCGGTTTTGAAGTTAATCTTTT (p) TGTGTTTCGGAGTGCTTT (VIC/MGB)	86	139	NC_006089.3
TNF-α	(f) CCCCTACCCTGTCCCACAA (r) ACTGCGGAGGGTTCATTCC (p) CTGGCCTCAGACCAG (VIC/MGB)	75	62	NC_006101.3
iNOS	(f) GAGTGGTTTAAGGAGTTGGATCTGA (r) TCCAGACCTCCCACCTCAAG (p) CTCTGCCTGCTGTTGCCAACATGCT (VIC/MGB)	103	80	NC_006106.3
LITAF	(f) TCTGAGACCCCCAAGTCCAA (r) CCTTAAGTTTTGCCAGAGGAGGTT (p) CCCACCACACCCACT (VIC/MGB)	98	197	NC_006104.3

IL-1β = interleukin 1 beta; IL-6 = interleukin 6; iNOS = inducible nitric oxide synthase; LITAF = lipopolysaccharide-induced TNF- α factor [43]; TLR-4 = toll-like receptor 4. f = forward primer; r = reverse primer; p = probe

### Statistical Analysis

For each age of blood sampling, data from the randomized design were subjected to an ANOVA [82] using the MIXED model procedure of the SAS Institute [83]. The main effect of treatment was fixed. The tier (deck) of cages was the experimental unit. Subsampling error terms included cages within tiers (2 cages per tier per treatment) and hens within cages within tiers (2 hens per cage per tier per treatment). Pooling of error terms occurred when *P* > 0.25. The data were normally distributed and reported as least square means ± SEM. Significant treatment effects were subjected to the SLICE option [84]. Significance was set at *P* < 0.05 for all statistical analysis.

## Results

Access of thermally cooled perches or ambient air perches did not affect body weight and weights of organs (the liver, spleen and right adrenal gland) of hens at 32 week of age followed a 16-week period of time during 2013 summer including a 4 h acute heat episode at 27.6 week of age (Table 2).

**Table 2:** The effect of thermally cooled perches on body and organ weights of 32-week-old White Leghorn hens^1^ in 2013 summer hot months including 31 days after an acute heating episode.

		Organ weights^2^					
Treatment	BW (g)	Spleen (mg)	Relative spleen (mg/100g of BW)	Liver (g)	Relative liver (g/100g of BW)	Right adrenal (mg)	Relative right adrenal (mg/100g of BW)
Cooled perch	1440 ± 31	1237 ± 78	86 ± 8	36 ± 2	2.5 ± 0.1	73 ± 5	5.0 ± 0.3
Non-cooled perch	1468 ± 31	1277 ± 78	88 ± 8	40 ± 2	2.6 ± 0.1	76 ± 5	5.1 ± 0.3
No perch	1456 ± 31	1178 ± 78	81 ± 8	35 ± 2	2.5 ± 0.1	73 ± 5	5.1 ± 0.3
n^3^	12	12	12	12	12	12	12
P-value	0.81	0.67	0.86	0.30	0.99	0.89	0.96

^1^ The data were collected during 2013 summer hot months including 31 days after an acute heating episode.

^2^Values represent least square Mean±SEM, n = 12.

^3^Number of observations per least square mean.

BW = body weight

There were no thermally cooled perch effects on the levels of PCV, circulating cytokines (IL-1β, IL-6 and LITAF) immediately after a 4 h acute heat stress (at 27.6 week of age) and followed a 16-week period of the summer season (at 32 week of age) (*P* > 0.05, respectively; Table 3). However, plasma concentrations of IgG were reduced in hens with accessed to cooled perches or air perches compared to control hens without accessed perches at 32 week of age (*P* < 0.005, Table 3) but not at 27.6 week of age (*P* > 0.05).

**Table 3:** The effect of thermally cool perch availability on PCV, plasma IgG and cytokine concentrations of White Leghorn hens at 27.6 and 32 week of age^1^.

Treatment	PCV (%)	IgG (mg/mL)	IL-1β (pg/mL)	IL-6 (mg/mL)	LITAF (pg/mL)
**27.6 week of age, during the 4 h heating episode**					
Cool Perch	28.8	6.92	1.19	0.93	144.48
Air Perch	28.2	6.94	0.92	1.05	143.40
No Perch	28.7	7.25	1.37	1.03	140.60
SEM	0.6	0.86	0.33	0.15	14.59
n^2^	12	12	12	12	12
P-value	0.76	0.84	0.63	0.83	0.50
**32 week of age, after 16 weeks of summer hot months with a 4 h heating episode**					
Cool Perch	28.3	15.79	3.64	1.31	190.67
Air Perch	27.4	14.67	3.70	1.02	194.20
No Perch	28.0	19.35*	3.85	0.89	188.75
SEM	0.5	1.57	0.73	0.25	4.53
n^2^	12	12	12	12	12
P-value	0.40	0.0053	0.98	0.50	0.70

^1^Data were measured using ELISA and presented as Mean±SEM.

*P < 0.05 compared with other treatments at 32 week of age.

^2^Average number of observations per least square mean at 27.6 and 32 week of age.

IL-1β = interleukin-1 beta; IL-6 = interleukin 6; LITAF = lipopolysaccharide-induced TNF- α factor [43]; PCV = packed cell volume.

There were no treatment effects on mRNA expression of splenic cytokines (IL-1β, IL-6 and LITAF), TLR-4, and iNOS at 32 week of age followed a 16-week period during the summer months including a 4 h acute heat episode at 27.6 week of age (*P* > 0.05, respectively; Table 4).

**Table 4:** The effect of thermally cool perch on mRNA expression of cytokines, toll-like receptors, and inducible nitric oxide synthase in the spleen of 32 week-old laying hens^1^.

Treatment	IL-1β	IL-6	LITAF	TLR-4	iNOS
Cool Perch	0.75	0.79	1.65	2.35	1.33
Air Perch	0.69	0.60	1.87	2.40	1.30
No Perch	0.68	0.59	2.10	2.04	1.04
SEM	0.11	0.15	0.57	0.56	0.27
n^2^	12	12	12	12	12
*P*-value	0.89	0.59	0.46	0.89	0.71

^1^Data were presented as Mean±SEM. The data were calculated by the equation: tested mRNA quantity value (mean of replicated target mRNA value/GAPDH quantity mean of replicate endogenous control value).

^2^Average number of observations per least square mean.

GAPDH = glyceraldehyde 3-phosphate dehydrogenase; iNOS = inducible nitric oxide synthase; IL-1β = interleukin 1 beta; IL-6 = interleukin 6; TLR-4 = toll-like receptor 4; LITAF = lipopolysaccharide-induced TNF-α factor [43].

**Fig 2:**
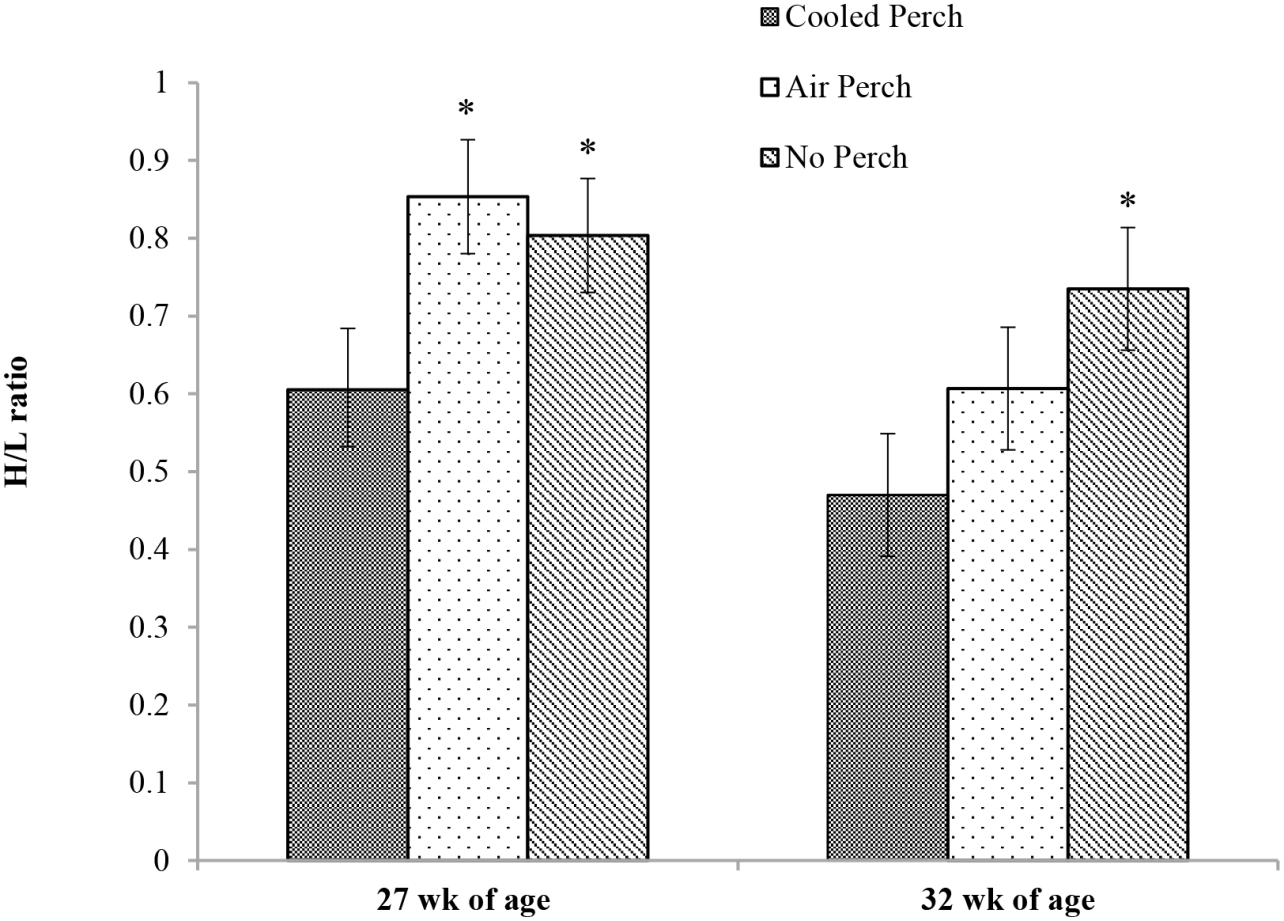
The effect of thermally cooled perches on heterophil/lymphocyte ratios. The heterophil to lymphocyte (H/L) ratios of hens subjected to a heating episode at 27.6 week of age and at the end of the study at 32 week of age. *Least square means were significant differences (*P* < 0.05) within the same age group.

Hens with access to cooled perches had a lower H/L ratio at 27.6 week of age, followed a 4 h acute heat episode (*P* < 0.01) compared to hens with access to air perches or control hens without perches. At 32 week of age, after a 16-week period of the summer season, the H/L ratios were still lower in hens with access to thermally cooled perches compared to control hens without perches (*P* < 0.05. Fig 2), but not to hens provided with air perches (*P* > 0.05).

## Discussion

High ambient temperature during summer months is one of the most important environmental stressors in the poultry industry. A chicken can die due to heat stress, especially hot waves, as Hegeman [6] reported that tens of thousands of turkeys and chickens in Kansas and North Carolina were killed in 2011 summer during a heat wave (over 100°F). For those hens that survive high temperatures, health profiles including immunity are detrimentally affected [1, 8, 85]. However, in the current study, compared to air perch and no perch hens, with the exception of the H/L response, thermally cooled perches during the summer months (June through September 2013) had little effect on hens’ (cooled perch hens) immune and physiological parameters as indicated by body weight; relative organ weights (the spleen, liver, and right adrenal gland); PCV; plasma levels of cytokines (IL-1ß, IL-6, and LITAF), and the mRNA expression of cytokines, TLR-4, and iNOS in the spleen. The mild summer of 2013 (a mean of 24°C ranged from 17.1 to 33.1°C) in West Lafayette, IN and the brevity of the acute heating episode (2–4 h at a mean of 33.3°C arranged from 32 to 34.6°C) were most likely the reasons for little change in these measured parameters of hens at both 27.6 and 32 week of age.

With regard to immune function, circulating catecholamines and corticosterone released by the adrenal glands that have undergone hypertrophy during stress bind to their receptors of immune cells [86] causing profound immunosuppression and lymphoid organ regression [87–89]. Heat stress reduces antibody production to specific antigens in chickens [8]. In the current study, however, cooled perch hens or air perch hens had lower natural IgG concentrations at 32 week of age but not at 27.6 week of age compared to that of control hens. Similar to the current results, Regnier et al. [18] reported that control hens had lower antibody titers than heat-stressed New Hampshire hens (36°C for 5 days). Although the cellular mechanisms of the different regulation of the hens’ IgG concentrations in the present study is unclear, it could mostly associate with perch usage as perch increasing skeletal health [77, 90] and decreasing social stress and its associated damage [91–92]. In supporting the hypothesis, Sun et al. [92] reported that the isotype titers of natural antibodies, antibodies without any previous antigen exposure, in laying hens were affected by environmental factors including row and level of the cages. Similar to the current results, a negative correlation between total IgG concentrations and productivity and survivability has been found in several previous studies, including body weight, feeding efficiency, and egg production [93–96]. In addition, Wondmeneh et al. [97] reported that chickens’ natural antibody titers were related to increased hazard in their housing environments.

The numbers of B- and T-lymphocytes, involved in antibody- and cell-mediated immunity, respectively, are reduced and their functions impaired under conditions of chronic stress [98]. Stressed-associated overexpressed glucocorticoid, as one of the reasons, induced lymphopenia, causing an increase in the H/L ratio which has been used as an indicator of stress [23, 99]. The H/L ratio was lower in cooled perch hens than both air perch and no perch hens at 27.6 week of age followed an acute heat episode; and the difference was extended to 32 week of age between cooled perch and no perch hens but not air perch hens. These results, cooled perch vs. air perch, indicate that acute heat episode (2–4 h at 33.3°C) caused a mild stress reaction but no stress response at 24°C (17.1 to 33.1°C) during the 16 wk period of 2013 summer season. The H/L ratio of hens at 32 week of age, cooled perch ≤ air perch < no perch, suggest that perches per se may have alleviated long-term stress, especially for cooled perch hens. Hens housed on a slat and littered floor with access to perches had lower H/L ratios than hens without perches suggesting that perches helped reduce stress [100], but a similar effect was not reported for caged hens with and without perches [101].

Although the results of the current study, H/L ratio, and a companion study using the same chickens, rectal temperature [102] and heat stress behaviors [103], provided some evidence that thermally cooled perches helped hens cope with acute heat stress, currently measured cytokines, IL-1β, IL-6, and LITAF, remained unaffected. Laying hens subjected to 12 d of HS (34°C) experienced an increase in serum levels of IL-1 and TNF-α [104], but the cooled perches of the current study were ineffective regulating the levels of these cytokines during a heating episode (33.3°C for 2–4 h period) even though rectal temperature was lower in the hens with perches as compared to hens without perches [102]. Similar to plasma cytokines, the splenic expression of cytokines (IL-1, IL-6, and LITAF), TLR-4, and iNOS at 32 wk of age was unaffected in hens with access to cooled perches. Other studies with chickens have reported an up-regulation of some of these immune parameters perhaps because the stressor was more severe [33]. The lack of a physiological response due to the presence of cooled perches as result of too mild of a stressful event including the acute heating episode, is further corroborated by the fact that egg production, shell quality, feed efficiency, foot health, and feather score [105], bone mineralization and muscle deposition [106] were unaffected by the cooled perch treatment.

However, a parallel behavioral observation showed that the hens exhibited heat-associated behaviors differently during the acute heating episode [103]. Specifically, perch use was significantly higher at all-time points in cooled versus air perch cages (F_1,10_ = 41.32, *P* < 0.0001) on the day of the acute heat stress event. The onset of panting and wing spreading was delayed in cooled perch cages as compared to air perch and no perch cages, and incidences of these behaviors remained lower within cooled perch cages as compared to air perch or no perch during and after the acute heating episode (*P* < 0.05, respectively). Furthermore, once panting and wing spreading was initiated in the cooled perch hens, the proportion of hens panting was always lower during and immediately following the 4-h heating episode compared to the air perch hens as well as the no perch hens. Interestingly, use of perches 3 d prior to the heating episode did not differ between hens provided thermally cooled as compared to air perches most likely because of mild summer weather [103]. These behavioral changes during the heating episode where hens sought out the cooled perches causing less panting and wing spreading helped them cope with the stressful heating event as exemplified by reduced rectal temperature as compared to hens with no perch but not the air perch [102]. Under HS, the method of birds to loss heat is shifted from sensible heat loss (radiation, conduction and convection) from surfaces (crown, wattles, shanks, and unfeathered areas under wings) to evaporative heat loss (panting). These results indicates that thermally cooled perch system used in this study effectively prevents acute heat episode induced stress reactions, however, 4 h heat wave at 33.3°C and natural ambient temperature of 2013 summer in Indiana were not severe enough to evoke physical and physiological changes. As suggested by Spencer [107], behavioral changes, such as panting and sweating, will occur long before physiological changes at less cost to the animal [108].

### Conclusions

In summary, the most significant change of immunological biomarker that responded to the thermally cooled perches after exposure to an induced heating episode of 4 h and 16 weeks of summer hot months was the reduced H/L ratio suggesting that these hens may be able to cope with acute heat stress more effectively than hens with air perches or without perches. Further studies are needed to evaluate the effectiveness of thermally cooled perches on hen health under higher ambient temperatures.

## Supporting Information

S1 Data The raw data of body weight and organ weights. (XLSX)

S2 Data The raw data of immune parameters. (XLSX)
