# Correction: The Effect of Cooled Perches on Immunological Parameters of Caged White Leghorn Hens during the Hot Summer Months

**DOI:** 10.1371/journal.pone.0152633

**Published:** 2016-03-25

**Authors:** Rebecca A. Strong, Patricia Y. Hester, Susan D. Eicher, Jiaying Hu, Heng-Wei Cheng

There is an error in Fig 1. Please view the corrected figure below.

**Fig 1:**
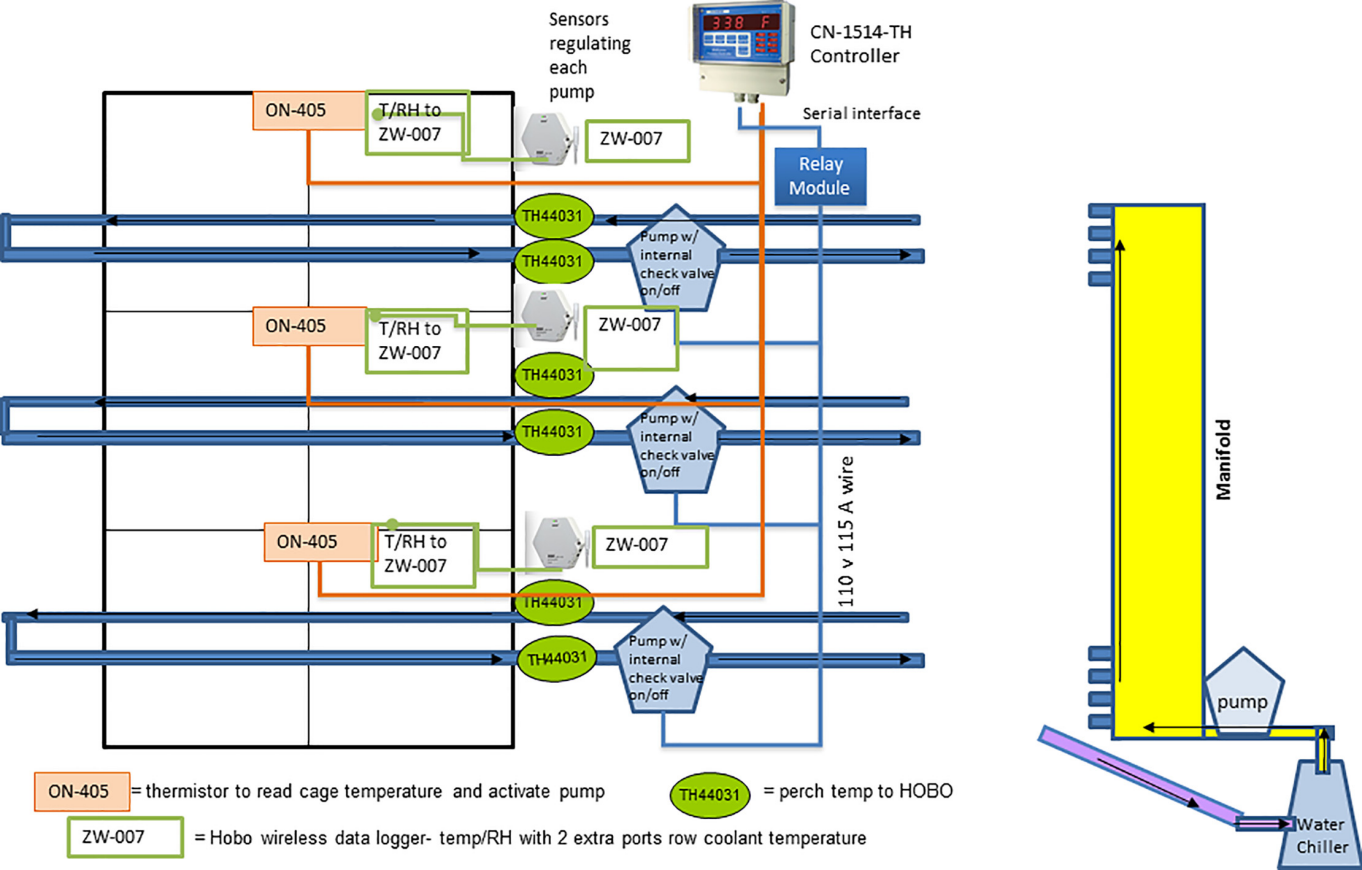
The bank of cages assignment for the thermally cooled perch treatment. Each tier had its own pump to distribute chilled deionized water (110°C) through its perch loop that ran parallel to the feeder. The front perch closest to the feeder received chilled water pumped directly from a common vertical manifold. The back perch was the return loop that sent the water back to the common manifold to be re-chilled. A chiller was used to cool the water in the manifold; it had its own independent thermostat which kept the water at 10°C. A separate 4th pump continuously circulated the deionized water between the water chiller and the manifold. A sensor for monitoring air temperature was installed to the controller of each tier to activate or stop the circulation of chilled water through the perch loop when ambient temperature reached or fell below 25°C, respectively [75].
